# Supplementary material for: Genome-wide DNA methylation profiling identifies convergent molecular signatures associated with idiopathic and syndromic autism in post-mortem human brain tissue
Source: Hum Mol Genet. 2019 Mar 8;28(13):2201–11. doi: 10.1093/hmg/ddz052 (PMC6602383; doi:10.1093/hmg/ddz052)
Supplement: Supp_ddz052 [file supp_ddz052.zip › Wong et al. ASD Brain Methylation_MS_SuppFigs_Revised_FINAL.docx]

**Supplementary Figures**

**Supplementary Figure 1: An overview of our study design.**

**
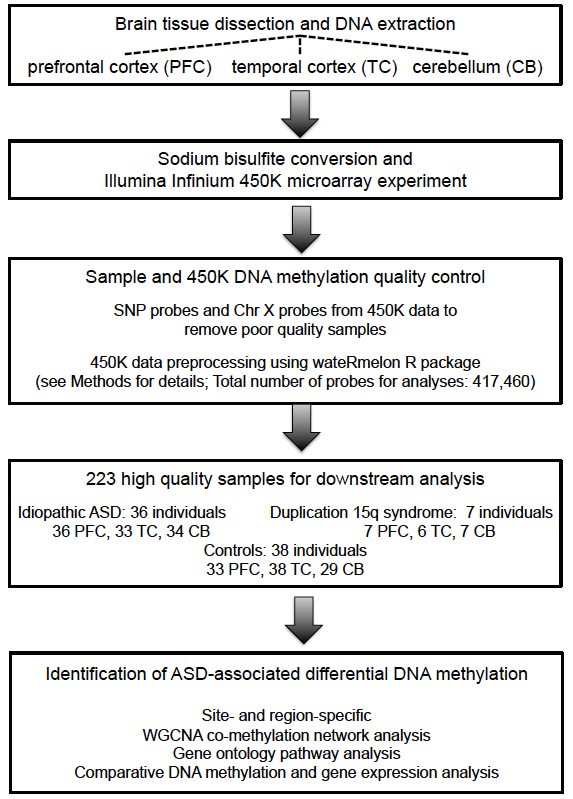
**

**Supplementary Figure 2: There is a strong positive correlation between the estimated ‘DNA methylation age’ - derived using an epigenetic clock based on DNA methylation values [1, 2] - and recorded chronological age for each donor across all three brain regions.** Shown are data for a) FC (r=0.98, P = 1.40e-52), b) TC (r=0.97, P = 5.69e-46), and c) CB (r=0.94, P = 9.43e-33). CTL = control samples, iASD = idiopathic autism cases, dup15q = 15q duplication carriers.

**Supplementary Figure 3: Hierarchical-clustering of the thousand most variable DNA methylation sites across all profiled brain samples.** Cerebellum samples are clearly distinct from the two cortical regions (prefrontal cortex (PFC) and temporal cortex (TC)). Cerebellum = purple, frontal cortex = blue, temporal cortex = green.

**
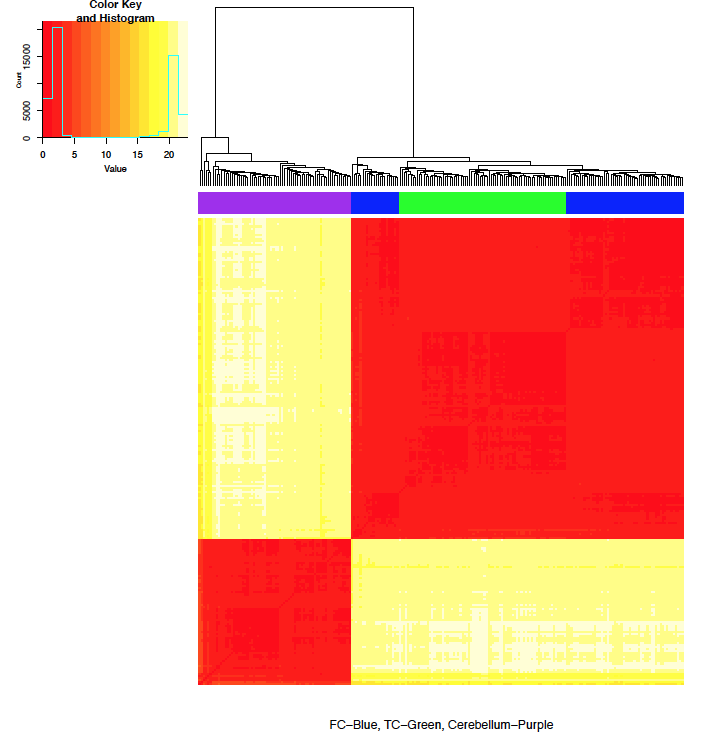
**

**Supplementary Figure 4: Effect sizes at iASD-associated DMPs are highly correlated between the two cortical regions (prefrontal cortex (PFC) and temporal cortex (TC) but not between cortex and cerebellum.** Shown are comparisons between tissues for **a)** the top 100 FC DMPs (FC vs TC: r = 0.77, P = 3.06e-21; FC vs CB: r = -0.14, P = 0.18), **b)** the top 100 TC DMPs (TC vs FC: r = 0.81, P = 2.48e-24; r = 0.17, P = 0.09), and **c)** the top 100 CB DMPs (CB vs FC: r = 0.005, P = 0.96; CB vs TC: r = -0.03, P = 0.77).


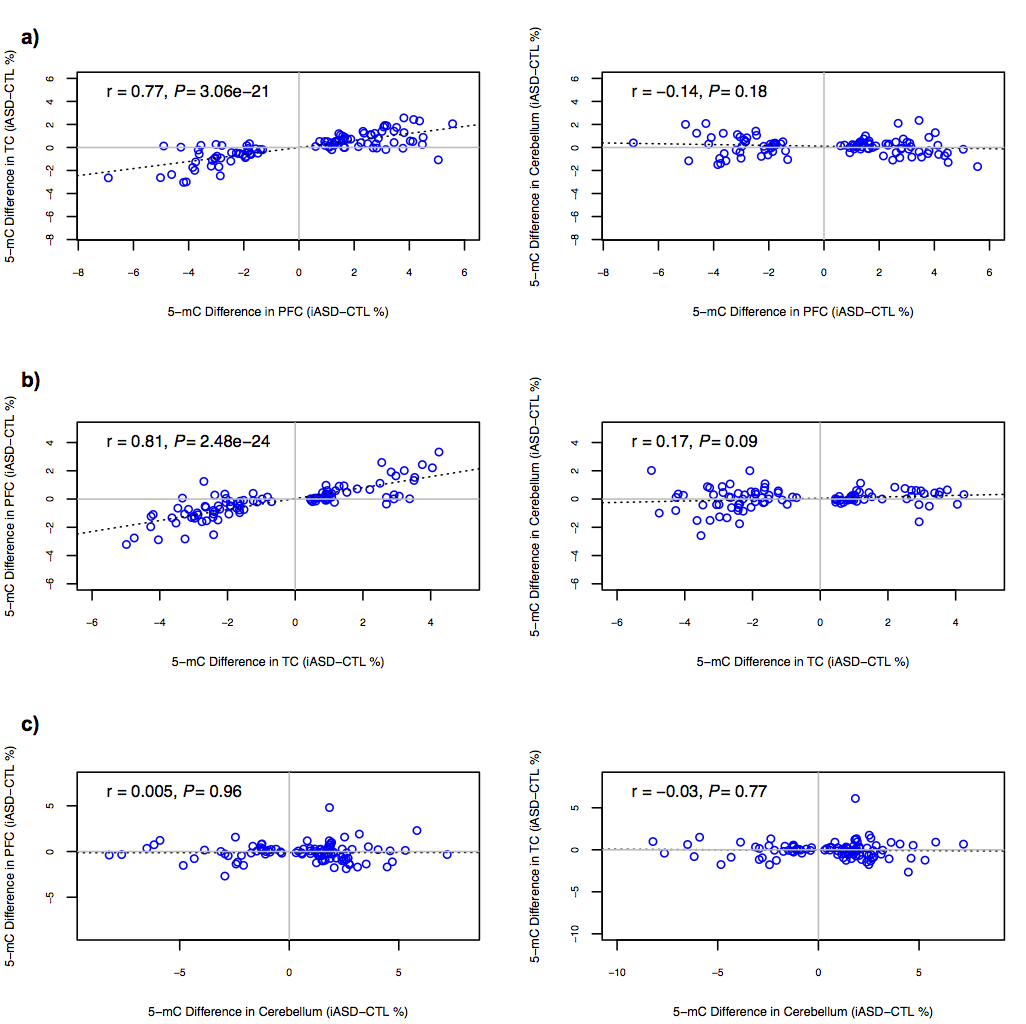


**Supplementary Figure 5: Manhattan plot showing P-values from a multi-level model used to identify consistent iASD-associated differences across both cortical regions (FC and TC).** We identified 157 DMPs (P< 5x10^-5^) with the top-ranked cross-cortex iASD-associated difference (cg14392966, P = 1.77E-08) being located immediately upstream of *PUS3* and *DDX25* on chromosome 11q24.2. *= P < 5 X 10^-5^.

**
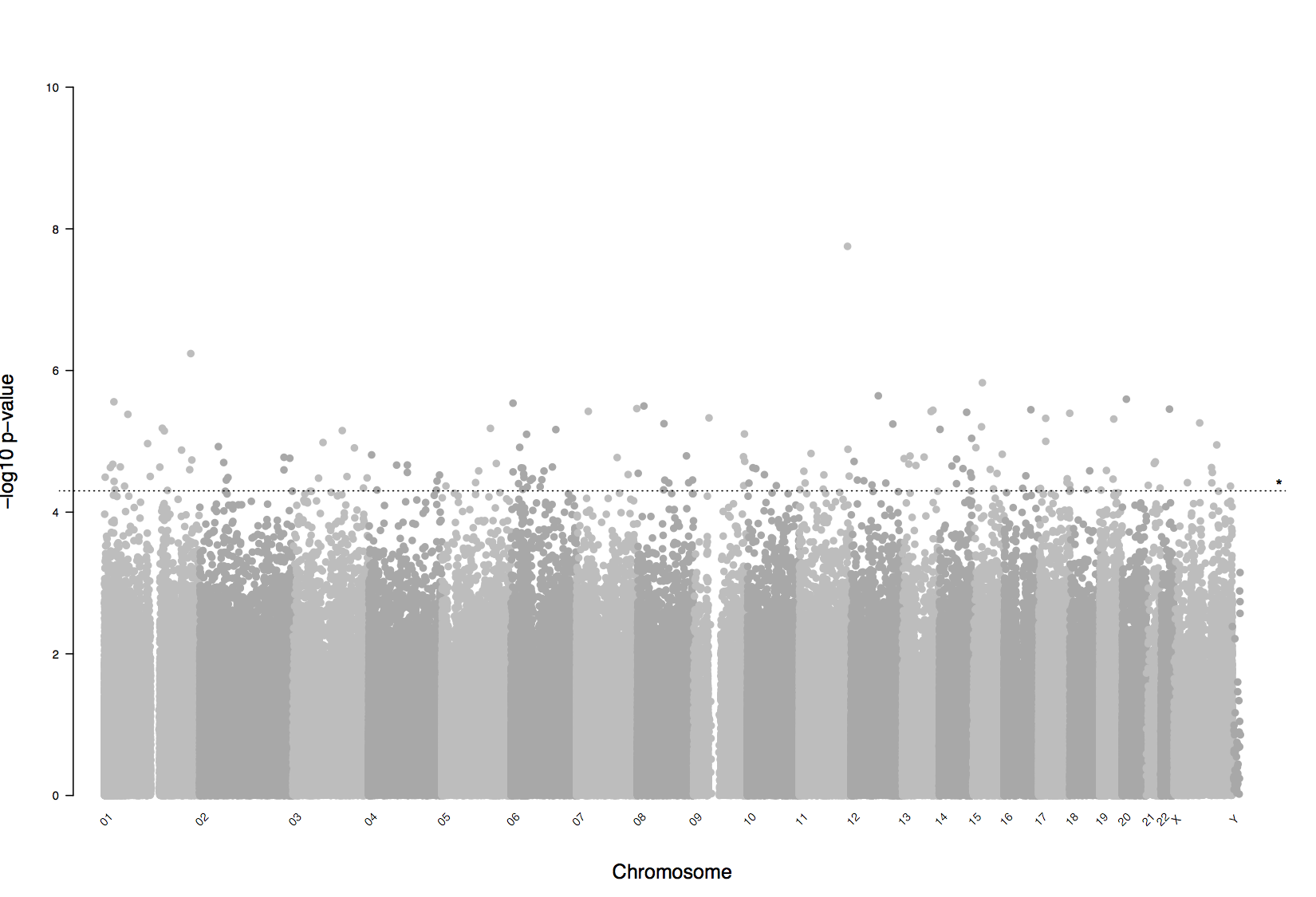
**

**Supplementary Figure 6. Dup15q-associated prefrontal cortex (PFC) DMPs are focused in a discrete differentially methylated domain within the duplicated region.** Shown is the distribution of P-values across the dup15q region from our dup15q vs. control analysis. Differentially methylated positions are stratified by direction of effect (red = hypermethylated in dup15q ASD, green = hypomethylated in dup15q ASD). Shown at the top are the estimated break-points for individual dup15q samples derived from DNA methylation data for each individual donor. The dup15q differentially methylated domain includes clusters of probes that are both hyper- and hypo-methylated overlapping a known imprinted gene cluster containing paternally-expressed (green), maternally-expressed (red) and biallelically-expressed (blue) genes.

**
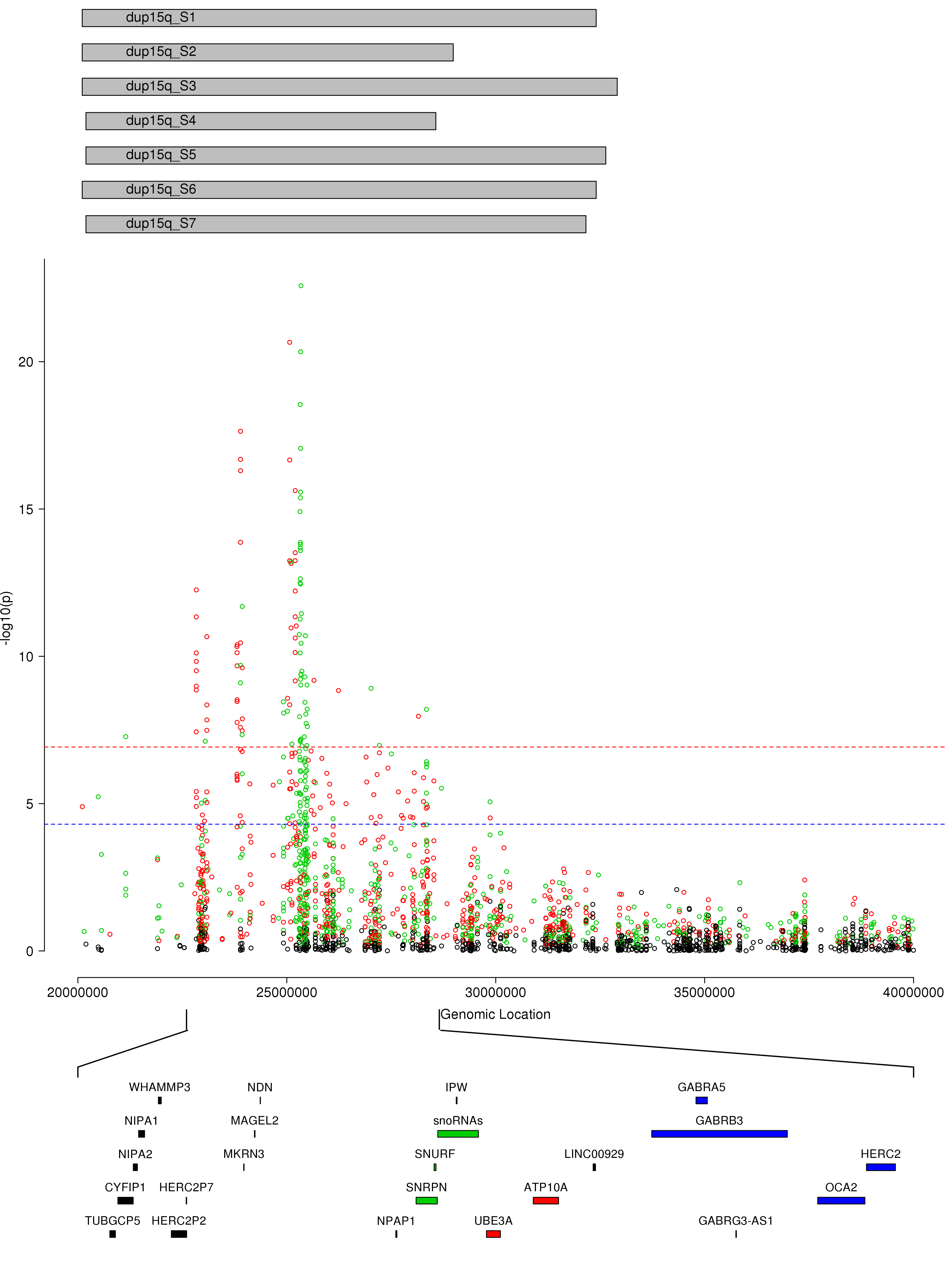
**

**Supplementary Figure 7. Dup15q-associated temporal cortex (TC) DMPs are focused in a discrete differentially methylated domain within the duplicated region.** Shown is the distribution of P-values across the dup15q region from our dup15q vs. control analysis. Differentially methylated positions are stratified by direction of effect (red = hypermethylated in dup15q ASD, green = hypomethylated in dup15q ASD). Shown at the top are the estimated break-points for individual dup15q samples derived from DNA methylation data. The dup15q differentially methylated domain includes clusters of probes that are both hyper- and hypo-methylated overlapping a known imprinted gene cluster containing paternally-expressed (green), maternally-expressed (red) and biallelically-expressed (blue) genes.

**
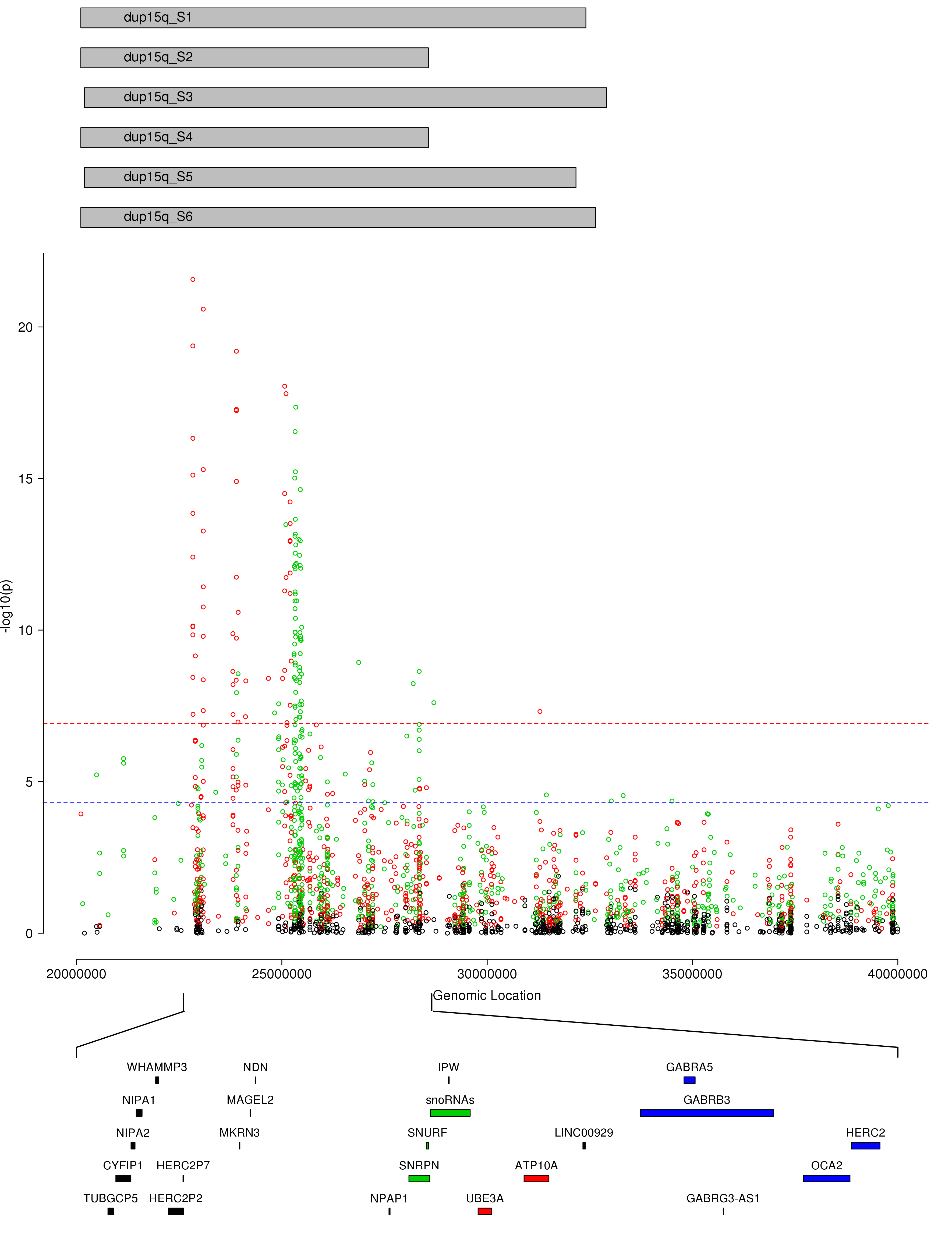
**

**Supplementary Figure 8. Dup15q-associated cerebellum DMPs are focused in a discrete differentially methylated domain within the duplicated region.** Shown is the distribution of P-values across the dup15q region from our dup15q vs. control analysis. Differentially methylated positions are stratified by direction of effect (red = hypermethylated in dup15q ASD, green = hypomethylated in dup15q ASD). Shown at the top are the estimated break-points for individual dup15q samples derived from DNA methylation data. The dup15q differentially methylated domain includes clusters of probes that are both hyper- and hypo-methylated overlapping a known imprinted gene cluster containing paternally-expressed (green), maternally-expressed (red) and biallelically-expressed (blue) genes.

**
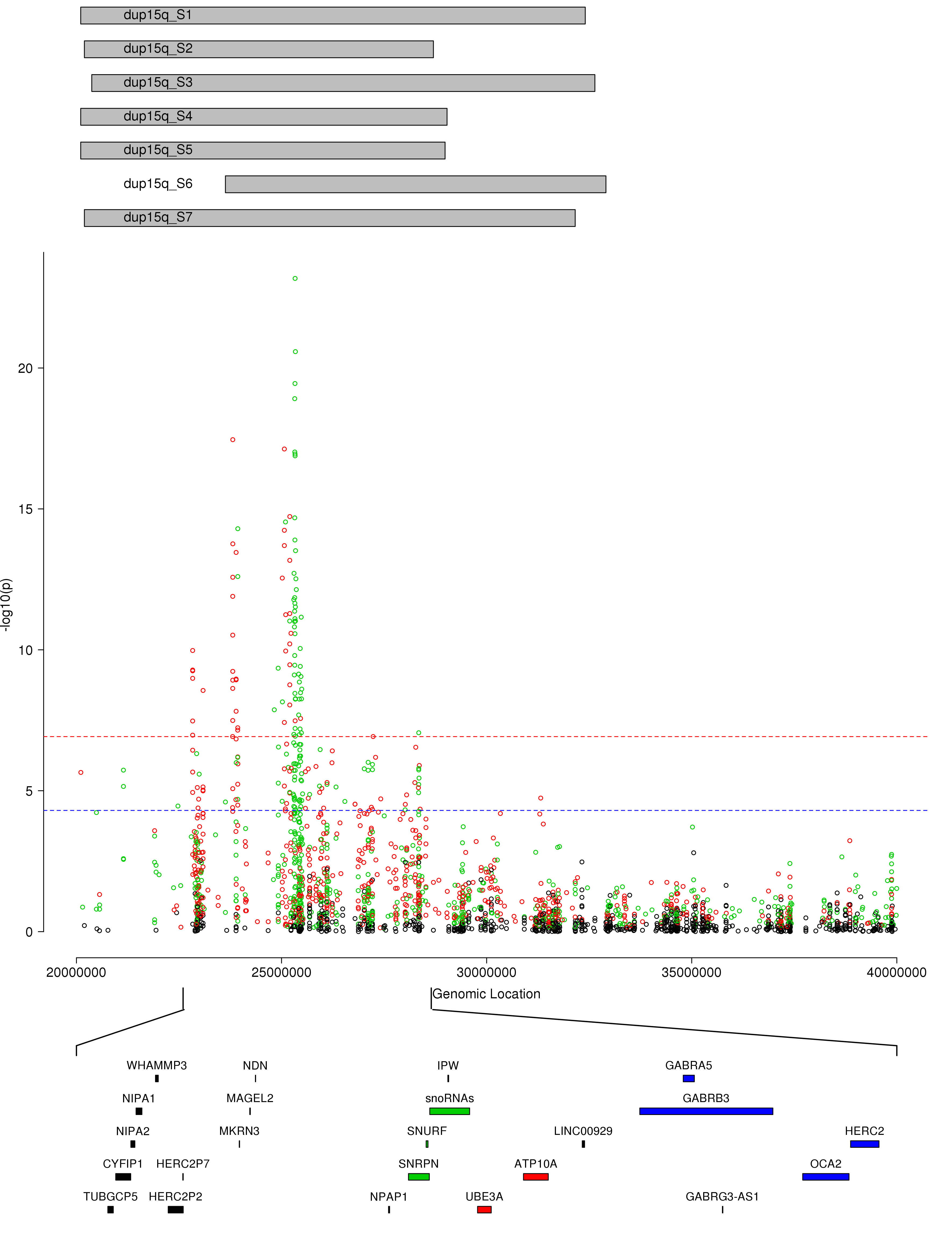
**

**Supplementary Figure 9: Manhattan plot of P-values from an analysis of differential prefrontal cortex (PFC) DNA methylation in dup15q carriers.** A list of significant dup15q-associated DMPs (**P < 1.198 x 10^-7^) are listed in **Supplementary Table 7**.

**
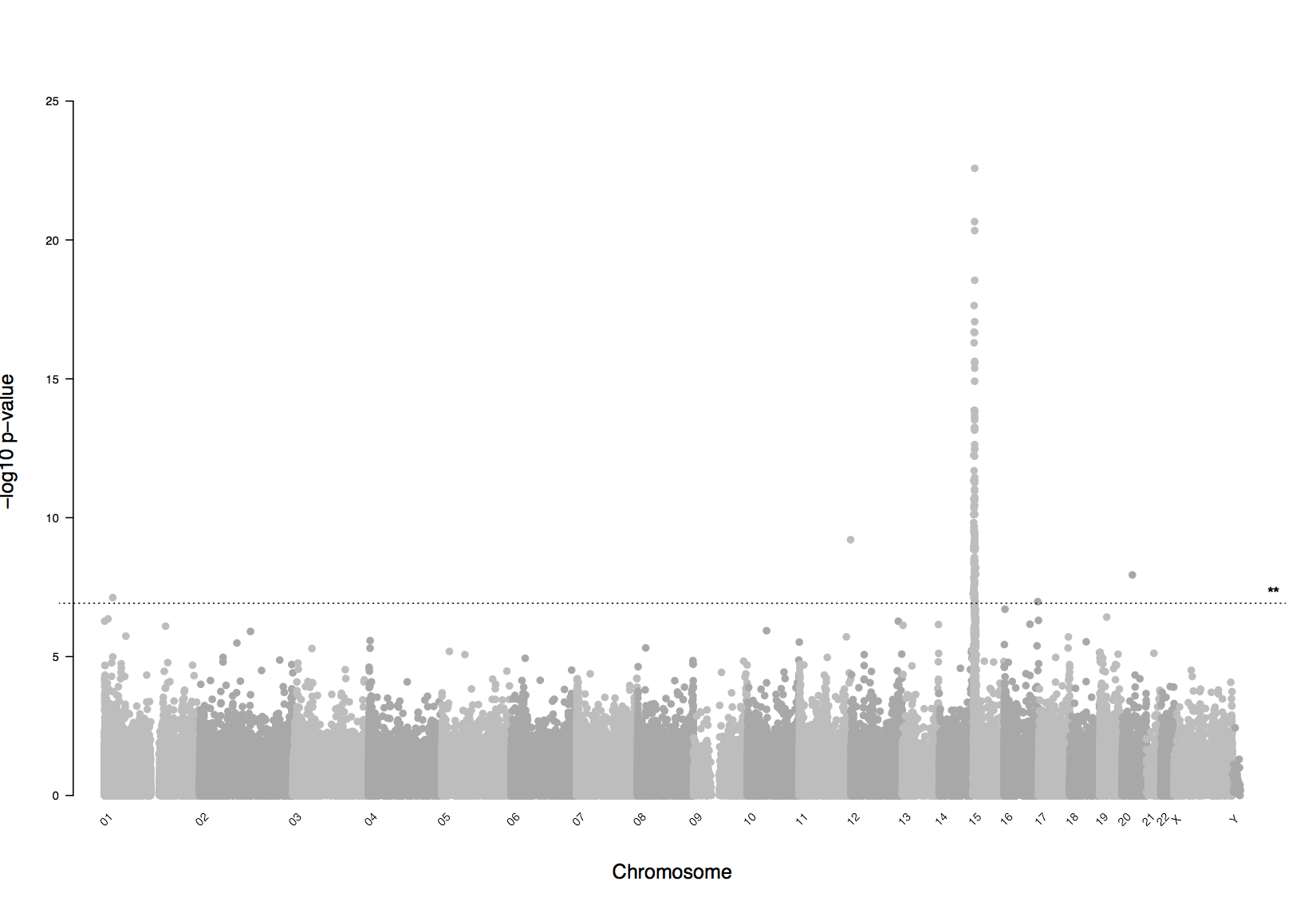
**

**Supplementary Figure 10: Manhattan plot of P-values from an analysis of differential temporal cortex DNA methylation in dup15q carriers.** A list of significant dup15q-associated DMPs (**P < 1.198 x 10^-7^) are listed in **Supplementary Table 8**.

**
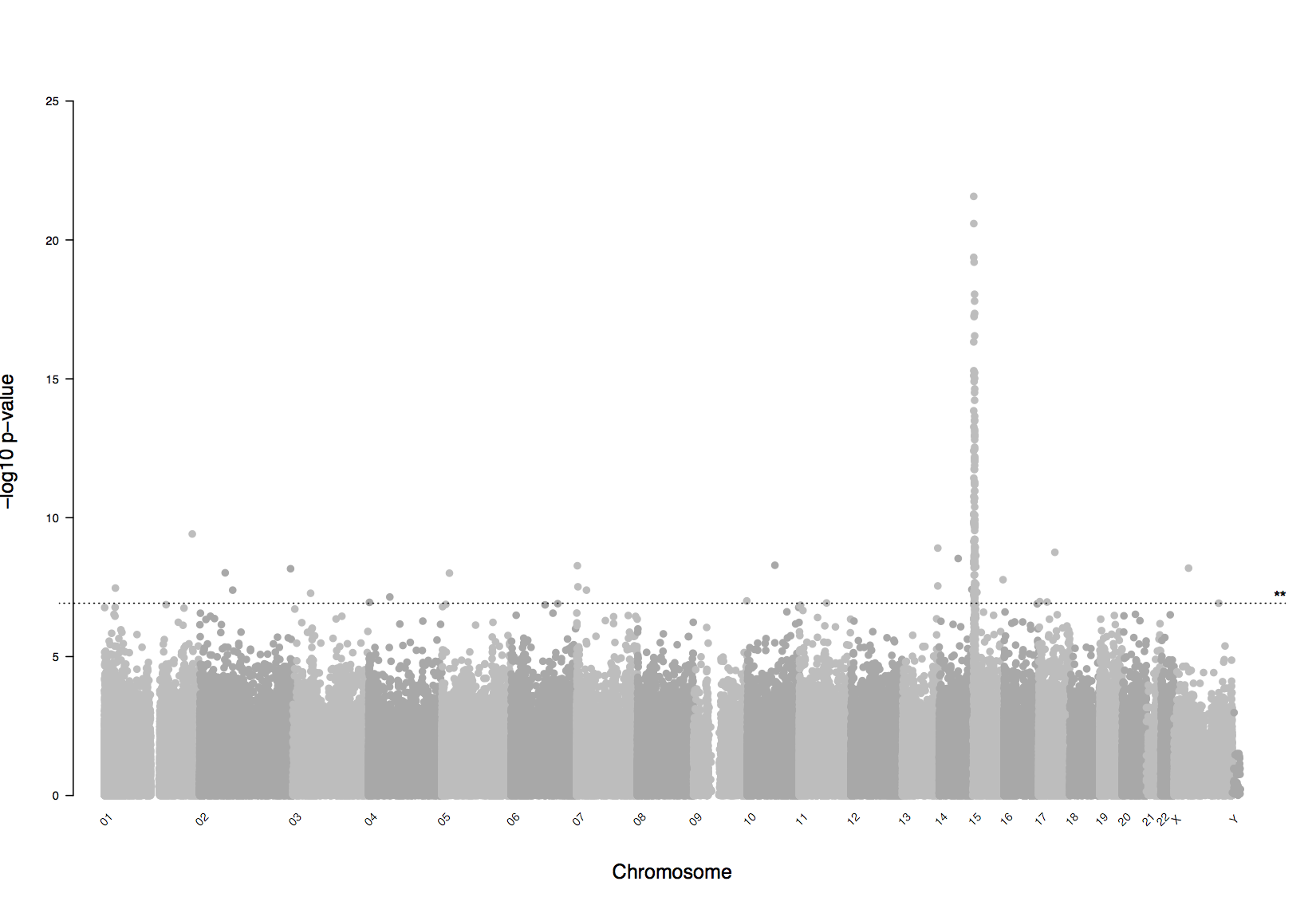
**

**Supplementary Figure 11: Manhattan plot of P-values from an analysis of differential cerebellum DNA methylation in dup15q carriers.** A list of significant dup15q-associated DMPs (**P < 1.198 x 10^-7^) are listed in **Supplementary Table 9**.

**
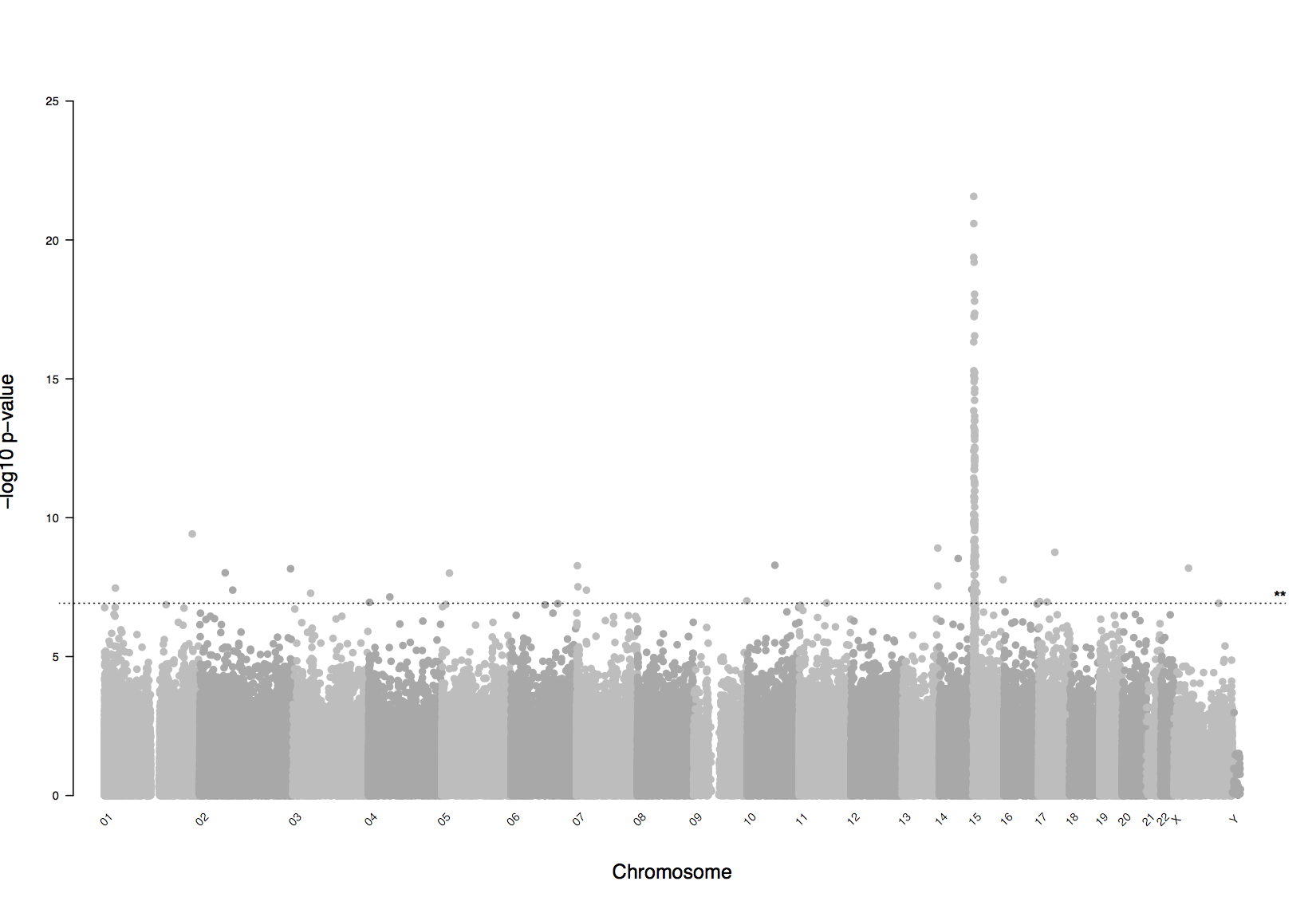
**

**Supplementary Figure 12: Although effect sizes at dup15q DMPs are highly consistent across each of the three brain regions, the overall pattern of dup15q-associated variation especially DMPs outside of dup15q regions are more similar between the two cortical regions (prefrontal cortex (PFC) and temporal cortex (TC) than between cortex and cerebellum (CB).** Shown are comparison between tissues for **A)** PFC DMPs with p< 5e-05 (PFC vs TC: across all probes r = 0.96, P = 9.73e-201, probes outside of dup15q regions r= 0.93, P = 1.13e-58; PFC vs CB: across all probes r = 0.87, P = 9.263-110, probes outside of dup15q regions r= 0.66, P = 4.95e-58), **B)** TC DMPs with p< 5e-05 (TC vs PFC: across all probes r = 0.79, P = <2.2e-16, probes outside of dup15q regions r= 0.73, P = 1.25e-221; TC vs CB: across all probes r = 0.64, P = 4.81e-177, probes outside of dup15q regions r= 0.44, P = 1.24e-62), and **C)** cerebellum DMPs with p< 5e-05 (CB vs PFC: across all probes r = 0.87, P = 5.44e-99, probes outside of dup15q regions r= 0.50, P = 7.40e-08; CB vs TC: across all probes r = 0.85, P = 1.81e-88, probes outside of dup15q regions r= 0.47, P = 4.02e-07).

**
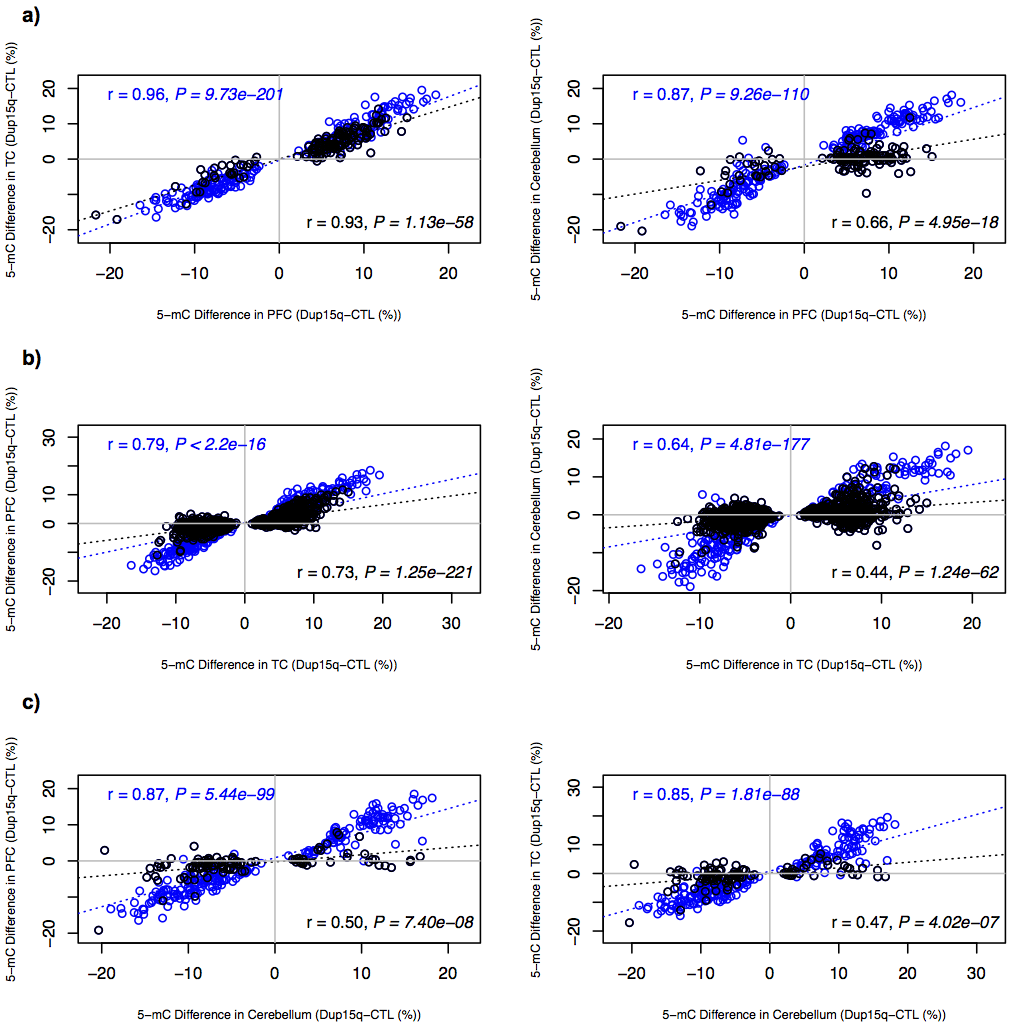
**

**Supplementary Figure 13: Hierarchical clustering of samples based on DNA methylation at iASD-associated DMPs in the prefrontal cortex (PFC).** Shown is the clustering of samples based on DNA methylation levels (red = low, yellow = high) at iASD-associated DMPs (P < 5e-05). iASD and dup15q samples are predominantly clustered together, separately to control samples.

**
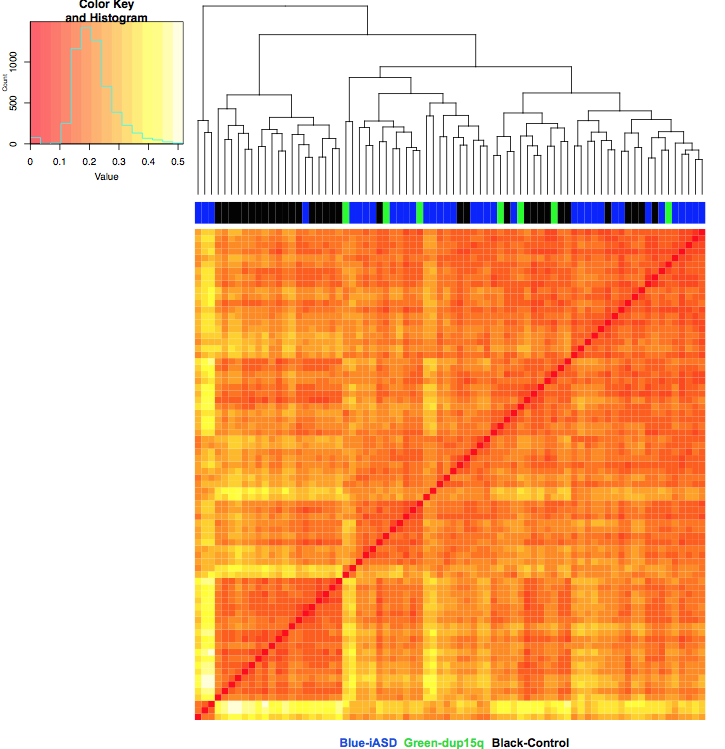
**

**Supplementary Figure 14: Hierarchical clustering of samples based on DNA methylation at iASD-associated DMPs in the temporal cortex (TC).** Shown is the clustering of samples based on DNA methylation levels (red = low, yellow = high) at iASD-associated DMPs (P < 5e-05). iASD and dup15q samples are predominantly clustered together, separately to control samples.

**
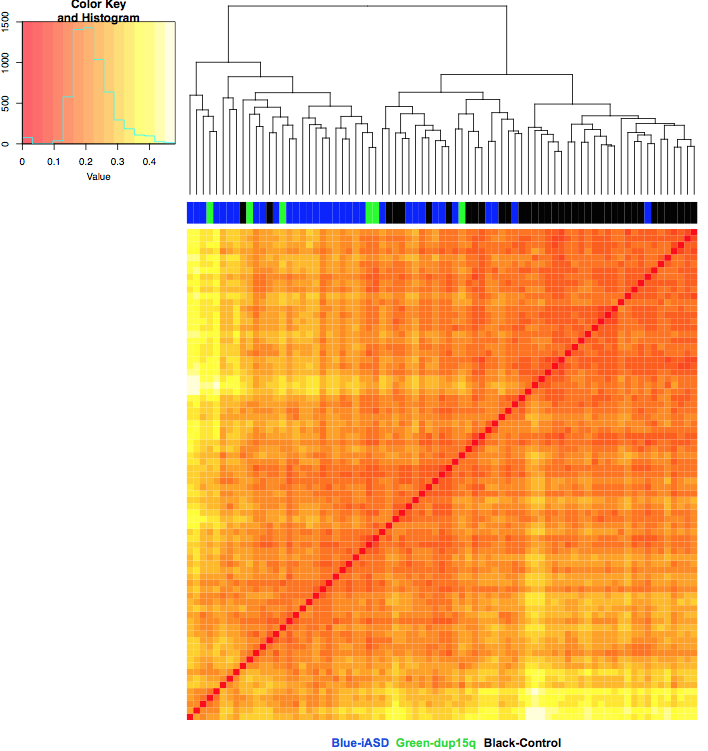
**

**Supplementary Figure 15:** **Autism-associated co-methylation modules.** Module plots displaying the top 15 hub DNA methylation sites (and their annotated genes) and top 50 connections for the eight co-methylation modules associated (FDR < 0.05) with at least one diagnostic category.

1. Horvath S: DNA methylation age of human tissues and cell types. *Genome biology* 2013, 14:3156.

2. Horvath S: Erratum to: DNA methylation age of human tissues and cell types. *Genome biology* 2015, 16:96.
